# Supplementary material for: A bireporter recombinant SARS-CoV-2 Omicron BA.5 for in vitro and in vivo studies
Source: Microbiol Spectr. 2026 Sep 10;14(10):e00217-26. doi: 10.1128/spectrum.00217-26 (PMC13689415; doi:10.1128/spectrum.00217-26)
Supplement: Supplementary file 1 [file spectrum.00217-26-s0001.docx]

**Supplementary table 1. Amino acid changes in rBA.5 ZsG-Nluc P6.**

| **Change** | **Coverage** | **Polymorphism Type** | **Allele Frequency** | **Variant P-Value (approximate)** | **Amino Acid Change** |
| --- | --- | --- | --- | --- | --- |
| C -> T | 566 | Single nucleotide (transition) | 0.94 | 0 | NSP9 (T21I) |
| G -> A | 1,317 | Single nucleotide (transition) | 0.72 | 0 | NSP14 (M58I) |
| T -> G | 1,341 | Single nucleotide (transversion) | 0.85 | 0 | S  (V971G) |
| -GTT | 227 | Deletion | 0.80 | 9.2E-233 | E  (ΔV14) |
| T -> A | 57 | Single nucleotide (transversion) | 0.35 | 6E-20 | N  (TRS) |
| T -> C | 37 | Single nucleotide (transition) | 0.43 | 1.8E-27 | N  (L13P) |
